# Warming-induced increase in carbon uptake is linked to earlier spring phenology in temperate and boreal forests

**DOI:** 10.1038/s41467-022-31496-w

**Published:** 2022-06-27

**Authors:** Hongshuang Gu, Yuxin Qiao, Zhenxiang Xi, Sergio Rossi, Nicholas G. Smith, Jianquan Liu, Lei Chen

**Affiliations:** 1 Key Laboratory of Bio-Resource and Eco-Environment of Ministry of Education, College of Life Sciences, Sichuan University, Chengdu, China; 2 Département des Sciences Fondamentales, Université du Québec à Chicoutimi, Chicoutimi, QC Canada; 3 Department of Biological Sciences, Texas Tech University, Lubbock, TX USA

**Keywords:** Phenology, Forest ecology

## Abstract

Under global warming, advances in spring phenology due to rising temperatures have been widely reported. However, the physiological mechanisms underlying the advancement in spring phenology still remain poorly understood. Here, we investigated the effect of temperature during the previous growing season on spring phenology of current year based on the start of season extracted from multiple long-term and large-scale phenological datasets between 1951 and 2018. Our findings indicate that warmer temperatures during previous growing season are linked to earlier spring phenology of current year in temperate and boreal forests. Correspondingly, we observed an earlier spring phenology with the increase in photosynthesis of the previous growing season. These findings suggest that the observed warming-induced earlier spring phenology is driven by increased photosynthetic carbon assimilation in the previous growing season. Therefore, the vital role of warming-induced changes in carbon assimilation should be considered to accurately project spring phenology and carbon cycling in forest ecosystems under future climate warming.

## Abstract (web summary)

The mechanisms underlying plant phenological shifts are debated. Here, based on phenological observations and ecosystem flux and climate data, Gu and colleagues provide evidence that warming-enhanced photosynthesis in a growing season contributes to earlier spring phenology in the following year in temperate and boreal forests.

## Introduction

Tree phenology influences not only the fitness and distribution of tree species, but also functioning of forest ecosystems, including water and energy fluxes and food web dynamics^1–4^. Changes in spring phenological events (e.g., bud-break and leaf-out) that indicate the start of growing season (SOS) are highly sensitive to temperature variation, especially in the extratropical regions^5^. Under global warming, an advanced spring phenology of trees (hereafter referred to as SOS) has been widely reported over recent decades due to rising temperature^6–8^. This advancement in SOS have been shown to extend the duration of the growing season and increase carbon uptake in forest ecosystems. Understanding the SOS in response to warming is therefore critical to assess the impacts of climate change on terrestrial carbon cycling and its feedbacks to climate^3,9^. However, the physiological mechanisms underlying the warming-induced advancements in SOS still remain poorly understood. This largely hinders the prediction of SOS and carbon cycling under future, warmer conditions.

In temperate and boreal forests, winter and spring temperatures are traditionally considered as the primarily driver of SOS because trees need to accumulate sufficient winter chilling units to end endodormancy and spring forcing units to break ecodormancy for reactivate growth^10–12^. Before entering dormancy, trees need to assimilate and store sufficient carbohydrates in the preceding growing season to resist the cold temperatures in winter and support growth reactivation in spring^13–15^. In temperate and boreal trees, nonstructural carbohydrate (NSC) concentrations, such as soluble sugar and starch, often reach maximum levels in autumn before winter dormancy, but become depleted by early summer after spring growth^16–18^. Experimental studies demonstrated that a later bud-break is often associated with a lower NSC availability in both broad-leaved and coniferous trees^16,19–21^. The SOS of current year is therefore likely to depend on the photosynthetic carbon assimilation during the previous growing season, yet this has received little attention across large spatial and temporal scales.

Under global warming, increasing temperatures may alter photosynthetic carbon assimilation, leading to changes in tree phenology^22^. Photosynthetic carbon uptake tends to show a peaked response to temperature at the leaf and canopy scale^23–26^. Collecting large-scale leaf-level photosynthesis experimental data across diverse biomes, Liang et al.^27^ showed that climate warming increased the net photosynthetic rate by 6.13% irrespective of an increased respiration. Using FLUXNET data, Duffy et al.^28^ demonstrated that the intersection point between photosynthesis and respiration is ~25 °C, and <10% of terrestrial biomes currently exceed this tipping point. As such, an increase in temperature during the previous growing season might increase photosynthesis in cold temperate and boreal regions, and advance SOS in the current year. However, previous studies have largely overlooked the links between previous growing season climate, photosynthesis, and the timing of SOS in the current year.

**Fig. 1:**
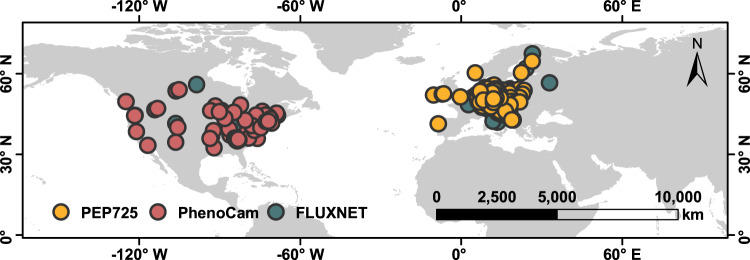
Distributions of the phenological observation sites in this study. Orange dots represent the 2322 sites selected from the PEP725 dataset across central Europe. Pink and green dots represent 67 sites in North America from the PhenoCam network and 28 FLUXNET sites, respectively.

Using long-term phenological observations, digital camera imagery, remote-sensing and flux data across temperate and boreal forests in the Northern Hemisphere (Fig. 1), we analyzed the effect of warming during the previous growing season on SOS. Here we show that timing of SOS in current year is advanced by warmer temperatures during previous growing season. Furthermore, we observe that SOS of current year occurs earlier with greater photosynthetic carbon assimilation in previous growing season. Our results provide evidence that warming-induced increase in carbon assimilation could be linked to the observed earlier SOS in temperate and boreal forests under climate warming.

## Results

Temperature sensitivity (S_T_, change in days per degree Celsius) is expressed as the slope of a linear regression between the dates of phenological events and the temperature. This approach has been widely applied to assess phenological responses to global climate warming^10,29,30^. To examine the effect of temperature during growing season (T_GS_) of previous year on SOS in temperate and boreal forests, we calculated the S_T_ using three complementary datasets (PEP725, PhenoCam, GIMMS NDVI_3g_). We first obtained normalized anomalies of SOS and T_GS_ at each site. Then, linear regression models were used to calculate S_T_ at each site separately, where the response variable was the normalized anomalies in SOS while the predictor was the normalized anomalies in T_GS_. Negative and positive S_T_ indicate advanced and delayed SOS by T_GS_, respectively.

**Fig. 2:**
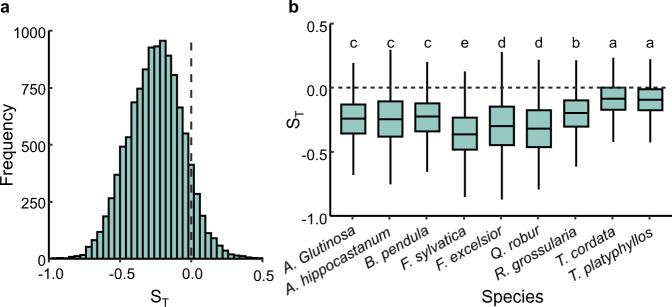
Temperature sensitivities (S_T_, change in days per degree Celsius) of start of season (SOS) in the current year in response to increasing temperature during previous growing season based upon PEP725 dataset. The calculated S_T_ was based upon records of spring leaf unfolding for 9 temperate tree species at 2322 sites in Europe. **a** Distribution of the calculated S_T_ across all species and sites (*N* = 11,369). **b** Difference in the S_T_ among tree species. The black dash lines indicate when S_T_ is equal to zero. The box spans from the first to the third quartile, with intermediate values marked as the black line in the middle of the box. One-way analysis of variance (ANOVA) followed by Tukey’s honestly significant difference (HSD) test was used to test the difference in the S_T_ between species, two-sided test was used to calculate *P* values and different letters indicate significant differences (*P* < 0.05). The sample size and calculated *P* values were listed in Supplementary Tables 2, 3.

Using the PEP725 dataset, we found that SOS was advanced by T_GS_ as indicated by the negative S_T_ across all selected nine temperate tree species (Fig. 2a). Among the tree species, the SOS response of *Fagus sylvatica* to T_GS_ was the strongest, and significantly higher than those of *Tilia cordata* and *Tilia platyphyllos* (Fig. 2b). To further ensure the robustness of our results, we calculated the partial correlation coefficients between T_GS_ and SOS after excluding co-variate effects of other climate variables, autumn leaf senescence, and chilling and forcing units (Supplementary Fig. 1). We observed a negative correlation between SOS and T_GS_. This also indicated that SOS was advanced by T_GS_.

**Fig. 3:**
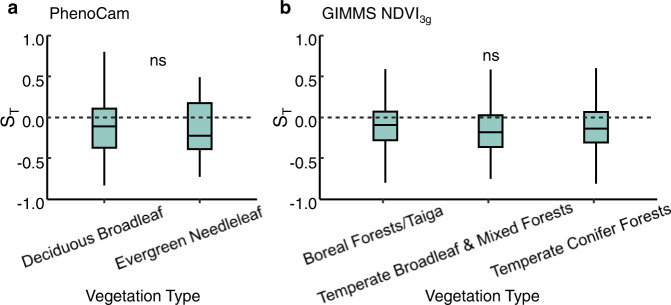
Temperature sensitivities (S_T_, change in days per degree Celsius) of start of season (SOS) in current year in response to increasing temperature during previous growing season. **a** The S_T_ based upon PhenoCam data in deciduous broadleaf and evergreen needleleaf forests. **b** The S_T_ based upon GIMMS NDVI3g data in boreal and temperate forests. The black dash lines indicate when S_T_ equals zero. In the box plots, the box spans from the first to the third quartile and the median is marked as the black line. One-way analysis of variance (ANOVA) followed by Tukey’s honestly significant difference (HSD) test was used to test the differences in the S_T_ between vegetation types, and two-sided test was used to calculate *P* values, the “ns” means no significant difference between groups (*P* > 0.05). The sample size and calculated P values were listed in Supplementary Tables 2, 3.

Our PEP725 results were corroborated by PhenoCam and remote-sensing data. Specifically, we observed a negative effect of T_GS_ on SOS in deciduous broad-leaved forests and evergreen forests using phenological metrics extracted from the PhenoCam network between 2000 and 2018 (Fig. 3a). Using the phenology metrics extracted from the remote-sensing dataset between 1982 and 2014, we also observed that increasing T_GS_ advanced SOS across different vegetation types in the Northern Hemisphere (Fig. 3b). However, no significant difference in S_T_ among vegetation types in PhenoCam and remote-sensing datasets was detected (Fig. 3a, b).

**Fig. 4:**
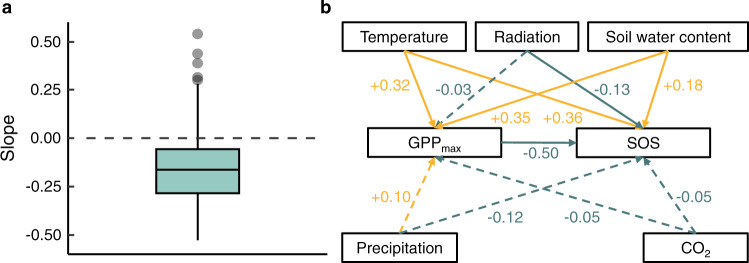
Effects of GPP_max_ and climate variables on start of season (SOS) based upon FLUXNET data. **a** Regression coefficients (Slope) of normalized anomalies of SOS in current year in response to normalized anomalies of GPP_max_ in previous growing season (*N* = 28). **b** Piecewise structural equation model (SEM) considering both GPP_max_ and climate variables. The GPP_max_ refers to the maximum daily gross primary productivity (GPP) in each year. In **a**, the black dash lines indicate when slope is equal to zero, the box spans from the first to the third quartile, with intermediate values marked as the black line in the middle of the box, and the gray points represent the outliers whose values exceed 1.5 times the length of the box. In **b**, both climate factors (temperature, radiation, soil water content, precipitation, and CO_2_) and GPP_max_ were incorporated into the SEM to explore the direct (arrows from each climate factor directly point to the SOS) or indirect (arrows from each climate factor firstly directly point to GPP_max_ then to the SOS) effects of climate factors on spring phenology, with green lines indicating a negative effect and orange lines indicating a positive effect. The solid lines represent significant relationships (*P* < 0.05) between variables, while dashed lines represent no significant relationships between variables (*P* > 0.05). The calculated *P* values based on two-sided test and other statistics were listed in Supplementary Table 4.

**Fig. 5:**
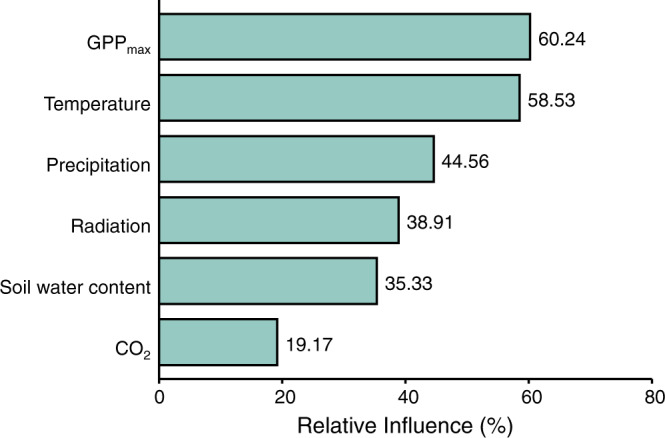
Relative influence of GPP_max_ and climatic factors during previous growing season on start of season (SOS) of current year using the data of 28 FLUXNET sites between 1992 and 2014. The GPP_max_ refers to the maximum daily gross primary productivity (GPP) in each year. Random-forest algorithm was used to quantify and compare the effects of climate variables and GPP_max_ on SOS.

Using the flux dataset, we found that the timing of SOS in the current year showed a significant negative correlation with the GPP_max_, the maximum daily gross primary productivity (GPP), during previous growing season between 1992 and 2014 (Fig. 4a). We also observed a significantly negative correlation between SOS in the current year and averaged GPP in previous growing season (Supplementary Fig. 2a). This suggested that spring phenology occurred earlier when photosynthetic carbon assimilation was greater during the previous growing season. To further test the carbon-based hypothesis, we constructed a piecewise structural equation model (SEM) to explore the direct and indirect effect of climate factors on SOS (Fig. 4b and Supplementary Table 4). We found that SOS was delayed directly by temperature and soil water content, but advanced by radiation in previous season. In addition, the direct effect of CO_2_ and precipitation on SOS was not significant (Fig. 4b). These direct effects are likely occurring due to spatiotemporal climate variation. We found that increased temperature and soil water content significantly increased GPP_max_. Then, SOS was significantly advanced by increased GPP_max_ (Fig. 4b), providing evidence for indirect effects of temperature and soil water content on SOS. We obtained similar results based on the averaged GPP in previous growing season (Supplementary Fig. 2b and Supplementary Table 5). Using random-forest analysis, we calculated and ranked the relative importance of GPP_max_ and other climatic drivers to SOS. Results showed that GPP_max_ and temperature were the strongest predictors of SOS, while relative importance of precipitation, radiation, soil water content and CO_2_ were lower (Fig. 5). Similar results were obtained when using averaged GPP during the previous growing season (Supplementary Fig. 3).

## Discussion

**Fig. 6:**
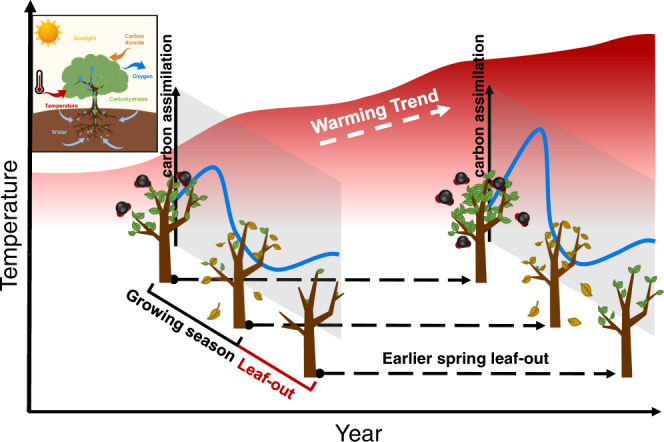
A schematic diagram of the earlier spring phenology in response to warming of previous growing season. Warmer temperatures during the previous growing season drivers earlier spring phenology by increasing photosynthetic carbon assimilation.

Global warming has advanced bud-break and leaf-out in temperate and boreal regions^6–8^. Using three long-term and large-scale phenological datasets, we show that warmer temperatures of the previous growing season led to earlier SOS of current year in temperate and boreal forests in the Northern Hemisphere. We also found that warming increased seasonal photosynthetic carbon assimilation, suggesting a physiological mechanism by which global warming is triggering earlier SOS in temperate and boreal forests (Fig. 6).

The carbon gained through photosynthesis can be stored in the form of nonstructural carbohydrates (NSC-soluble carbohydrates and starch), in part to support the growth of buds and leaves in the following spring^13–15^. For instance, 95% of the starch stored in the branches of *Fagus sylvatica* and *Quercus petraea* were consumed during bud break^31^. Needle growth of *Larix gmelinii* in spring drew nearly 50% of the carbohydrates fixed in the previous year^32,33^. The starch stored in 1-year-old needles of *Picea abies* and *Pinus sylvestris* exhibited a significant decline when needle production was complete^34^. Trees need to store sufficient carbohydrates before the winter dormancy period to maintain baseline functions and protect cells from frost damage and ensure survival in temperate and boreal regions^35,36^. Phloem girdling experiments showed that deficient carbon storage can delay SOS in both deciduous broad-leaved and evergreen coniferous trees^16,19^. Therefore, warmer temperatures in the previous growing season may advance SOS of current year by increasing carbon storage, supported by the negative correlations we observed between SOS of current year and GPP of previous growing season. To further test the carbon-driven hypothesis, we constructed a carbon-based SEM model to analyze the relationships between climate factors, GPP and spring phenology. We found that climate has both a direct effect on phenology, as has been shown previously^6,37^, but also an indirect effect through GPP. This again suggested that warming in the previous growing season may influence SOS by altering the photosynthetic carbon assimilation.

Recently, Zani et al.^22^ has reported that increased carbon assimilation during the growing season drives earlier autumn leaf senescence in temperate ecosystems. When leaf senescence occurred earlier, trees advanced endodormancy, and the requirement of chilling units may be also fulfilled earlier. As a result, earlier autumn phenology facilitates an earlier spring phenology^38,39^. Therefore, increased carbon assimilation may directly drive autumn phenology, and, in turn, influence spring phenology. Furthermore, spring phenology is also influenced by chilling and forcing units in winter and spring^29,40,41^. After excluding the co-variate effect of other climate factors in growing season, autumn phenology, chilling and forcing units on leaf unfolding, we also observed that SOS in current year was advanced by increasing temperature during the previous growing season. This provided additional support for our hypothesis that increased carbon assimilation in the previous season triggers an earlier spring phenology.

Using the PEP725 data, we observed a significant difference in the S_T_ among temperate tree species, which may be related to the species-specific photosynthetic leaf trait differences. For example, maximal carboxylation rate (V_cmax_) and maximal photosynthetic electron transport rate (J_max_) of *Quercus robur* and *Fraxinus excelsior* increased faster than *Betula pendula* with increasing temperature^42^. Correspondingly, *Quercus robur* and *Fraxinus excelsior* also showed a stronger phenological response to warming than *Betula pendula*. Using PhenoCam and remote-sensing data, we compared the difference in the S_T_ between deciduous broad-leaved and evergreen conifer forests, but no significant difference was observed between them. Compared with evergreens, the leaf life-span is shorter in deciduous species, and thus the time window allowed for photosynthesis is shorter^43,44^. However, deciduous species usually hold a larger leaf size and higher photosynthetic capacity than evergreens^45,46^. This could compensate for their shorter leaf life-span, and may partly explain the observed similar phenological responses between deciduous and evergreen forests.

Despite the warming-induced spring phenology observed in temperate and boreal regions, the underlying causes and physiological mechanisms still remain unclear. Using multiple long-term and large-scale datasets, herein we demonstrated that an increase in carbon assimilation under global warming could be involved in the observed earlier spring phenology in temperate and boreal forests. Our study provides new insights into the warming-induced change in spring phenology under global climate change to predict spring phenology and vegetation-atmosphere feedbacks under future climatic scenarios.

## Methods

### PEP725 phenological network

Phenological observation data were acquired by the European phenology database PEP725 (http://www.pep725.eu/). PEP725 contains phenological observations of temperate species across central Europe starting in 1951^47^. We selected the date when the first leaf stalks were visible (BBCH11 in PEP725) to represent SOS and date when 50% leaves had their autumnal color (BBCH94 in PEP725) to represent the end of growing season (EOS). Data exceeding 2.5 times of median absolute deviation (MAD) were considered outliers and removed^48^. We selected 466,988 records of nine temperate tree species (Supplementary Table 1) at 2322 sites, for a total of 171,202 species-site combinations with at least 30 years of observations.

### PhenoCam network

The PhenoCam network (https://phenocam.sr.unh.edu/) is a cooperative database of digital camera imagery that provides the dates of phenological transition between 2000 and 2018 across a diverse vegetations in Northern America^49^. In the PhenoCam network, the 50%, 75% and 90% of the Green Chromatic Coordinate (G_CC_) were calculated daily to extract the date of greenness rising and falling based on the following formula:

1 $${{{\mbox{G}}}}_{{{\mbox{CC}}}}\,=\,\frac{{{{\mbox{G}}}}_{{{\mbox{DN}}}}}{{{{\mbox{R}}}}_{{{\mbox{DN}}}}\,+\,{{{\mbox{G}}}}_{{{\mbox{DN}}}}\,+\,{{{\mbox{B}}}}_{{{\mbox{DN}}}}},$$

where *R*_*DN*_, *G*_*DN*_, and *B*_*DN*_ are the average red, green and blue digital numbers (*DN*), respectively.

We selected 50% amplitude of G_CC__90 (G_CC_ reaches 90th quantiles of its seasonal amplitude) as SOS^50^. We removed outliers when exceeding 2.5 times of MAD, and selected sites with at least 8-year observations between 2000 and 2018. Because we restricted study area to temperate and boreal forests, we excluded non-forest sites in the PhenoCam dataset. The final dataset had a total of 67 sites across deciduous broadleaf and evergreen coniferous forests.

### GIMMS NDVI_3g_ phenological product

The Normalized Difference Vegetation Index (NDVI), a proxy of vegetation greenness and photosynthetic activity, is commonly used to derive phenological metrics^51^. We derived SOS from the third generation GIMMS NDVI_3g_ dataset (http://ecocast.arc.nasa.gov) from Advanced Very High Resolution Radiometer (AVHRR) instruments for the period 1982–2014 with a spatial resolution of 8 km and a temporal resolution of 15 days^52^.

We only kept areas outside tropics (latitudes >30°N) that have a clear seasonal phenology^53^ and excluded bare lands with annual average NDVI < 0.1 to reduce bias. We applied a Savizky-Golay filter^54^ to smooth the time series and eliminate noise of atmospheric interference and satellite sensor, and used a Double Logistic 1st order equation to extract phenology dates^53^ according to the formula:

2 $$\text y(t)\,=\,a\left(\frac{1}{1{{\mbox{+}}}{{{\mbox{e}}}}^{{{\mbox{k(t-m)}}}}}{{\mbox{+}}}\frac{1}{1{{\mbox{+}}}{{{\mbox{e}}}}^{{{\mbox{e(t-n)}}}}}\right){\text+\,{b}},$$

where a, *k*, *m*, and *n* are parameters of logistic function and a is the initial background NDVI value, a + *b* represents the maximum NDVI value, *t* is time in days, and y(*t*) is the NDVI value at time *t*. The second-order derivative of Eq. 2 was calculated to extract SOS and EOS at the first and second local maximum point, respectively^55,56^. We excluded tropical and subtropical forests, and non-forest vegetation types based on a map of terrestrial ecoregions and focused on northern temperate and boreal forests^57^. The selected forest biomes include Boreal Forests/Taiga, Temperate Conifer Forests, and Temperate Broadleaf and Mixed Forests.

### FLUXNET dataset

The flux dataset was downloaded from FLUXNET (https://fluxnet.org/data/). The FLUXNET-2015 dataset was released in November 2016 (Total: 212 sites worldwide)^58^. The FLUXNET-2015 dataset provides data at different time scales, including half-hourly, daily, weekly, and yearly. The data at daily, weekly, and yearly scales were generated based on the original half-hour data using a data processing pipeline^58^, which was applied to reduce uncertainty by improving the data quality control and generate uniform and high-quality derived data products suitable for studies that compare multiple sites^58^. To be consistent with the temporal resolution of PEP725 and PhenoCam data, we selected daily-scale data from the FLUXNET-2015 dataset. Because we focused on temperate and boreal forests in the Northern Hemisphere, we selected a total of 28 forest sites with at least 10-years of observations and >300 daily records per year between 1992 and 2014 in the Northern Hemisphere. Here, we used mean GPP and GPP_max_ (the maximum daily GPP) during previous growing season to evaluate the photosynthetic carbon fixation at the ecosystem scale^59–61^. Singular Spectrum Analysis (SSA) filter method was first used to smooth the time series of daily GPP to minimize the noise^62,63^. The annual GPP_max_ was then obtained by extracting the maximum daily GPP values in each year from the smoothed GPP curve. The average GPP was calculated as the mean daily GPP during growing season between May and September. The SOS was extracted from smoothed daily GPP curve based on the threshold method^54^. The spring threshold was defined as 15% of the multi-year daily GPP maximum following previous studies^64,65^, and SOS was defined as the turning point when the smoothed GPP was higher than spring threshold.

### Climate data

Gridded daily mean temperature (°C), solar radiation (Wm^−2^), air humidity (%) and daily total precipitation (mm) during 1950–2015 in Europe were downloaded from the database E-OBS (http://www.ecad.eu/)^66^ at 0.25° spatial resolution. Gridded monthly soil moistures (kg/m^2^) during 1979–2015 were downloaded from World Meteorological Organization (http://climexp.knmi.nl/select.cgi?id=someone@ somewhere&field=clm_wfdei_soil01) at 0.5° spatial resolution and banded with PEP725 dataset. Global monthly mean temperatures during 1981–2017 were downloaded from Climate Research Unit (https://crudata.uea.ac.uk/cru/data/hrg/cru_ts_4.04/; http://www.geodata.cn) at 0.5° spatial resolution to match the PhenoCam and GIMMS NDVI_3g_ datasets. Bilinear interpolation method was used to extract climate data of each site or pixel using the “raster” package^67^ in R^68^. Environmental variables, including daily mean temperature (°C), shortwave radiation (Wm^−2^), CO_2_ (ppm), and precipitation (mm) were also extracted from the FLUXNET dataset. The detailed information of all the datasets used in our study was listed in Supplementary Table 6.

### Statistical analyses

The phenological records from PEP725 network are direct observations in the field, have a higher quality, and cover a longer period (1951–2015). However, most sites of PEP725 network are located in Central Europe and constrained to a relatively small spatial scale. In contrast, the extracted phenological metrics from PhenoCam images and remote-sensing products cover a large spatial scale regardless of their shorter periods. Therefore, we combined them together when performing statistical analyses to ensure precision and representativeness of our results.

We first calculated the temperature sensitivity (S_T_, change in days per degree Celsius) based on mean temperatures during the previous growing season (T_GS_) and timing of SOS using the three complementary datasets (PEP725, PhenoCam, GIMMS NDVI_3g_) in the Northern Hemisphere. The S_T_ was defined as the slope of linear regression between the dates of phenological stages and the temperature^10,29,30^. The mean dates of SOS and EOS from the PEP725 network were DOY 120 and DOY 280. Therefore, the period between May and September was selected to represent the growing season. A similar time period was used for complimentary analyses of PhenoCam and GIMMS NDVI_3g_ data. To calculate the S_T_, we first obtained normalized anomalies of SOS and T_GS_ relative to their long-term average at each site or pixel. Then, linear regression models were used to calculate S_T_ at each site or pixel separately, in which the response variable was the normalized anomalies in SOS while the predictor was the normalized anomalies in T_GS_. One-way analysis of variance (ANOVA) followed by Tukey’s honestly significant difference (HSD) test was used to test the difference in the S_T_ between species and vegetation types.

In addition to temperature, other climate factors (e.g., radiation and precipitation) may influence SOS by altering leaf photosynthesis. Partial correlation analysis has been frequently used to exclude confounding effects in order to isolate the relationship between two variables^69^. Using partial correlation analysis, here we excluded potential co-variate effects of a set of climate variables of growing season, including radiation, precipitation, soil moisture, humidity, on SOS and further examined the relationship between T_GS_ and SOS in PEP725 network. Because radiation and soil moisture data were only available since 1980, we selected phenology and climate datasets between 1984 and 2015.

Commonly, temperatures in winter and spring act as the dominant driver of spring phenology by influencing the accumulation of chilling and forcing units^29,37^. Importantly, effect of autumn phenology on spring phenology has been also reported^38,39^. To ensure the robustness of results, therefore, we also calculated the partial correlation coefficient between T_GS_ and SOS after excluding the co-variate effects of EOS and accumulations of chilling and forcing units, respectively, to test the relationship between T_GS_ and SOS in PEP725 network. Accumulations of chilling and forcing units were calculated during the period between November 1st and mean SOS across years for each species at each site^10^. The chilling units were calculated as the number of chilling days when daily mean temperature ranged from 0 to 5 °C; and the forcing units were calculated as the accumulated growing degree days when the daily mean temperature was above the threshold temperature 5 °C^10,70^. We performed partial correlation analysis using “ppcor” package^71^ in R^68^.

To clarify the underlying physiological mechanisms, we further examined the relationships between GPP (both average GPP and GPP_max_) of the previous growing season and SOS between 1992 and 2014 using FLUXNET data. Because the site-averaged daily GPP from FLUXNET started to increase from DOY 120, peaking at DOY 180, then decreased until DOY 300, the period between May and September was also selected as the growing season for calculating the climate variables. This is also consistent with the period of growing season identified by the dates of leaf unfolding and leaf senescence in PEP725 network. To examine the relationship between SOS and average GPP_max_, we first obtained normalized anomalies of SOS and GPP_max_ relative to their long-term average at each site. Then, linear regression models were applied to analyze the relationship between GPP_max_ anomalies on SOS anomalies for each site separately. In the models, the response variable was the normalized anomalies in SOS of current year and the predictor was normalized anomalies in GPP_max_ of previous growing season. The 95% confidence interval of the site-level regression slopes was calculated to determine the statistical significance of the relationship between GPP anomalies and SOS anomalies. The same analysis was repeated for average GPP instead of GPP_max_.

We further used piecewise structural equation models (SEM) to analyze the relationships between climate, GPP (both average GPP and GPP_max_) and SOS from the FLUXNET sites^72^. To test our carbon-based hypothesis, we constructed a conceptual model that includes both the direct and indirect effects of climate factors in growing season on spring phenology. In the SEM model, we hypothesized that climate during the growing season is likely to directly influence the timing of spring phenology, i.e., plants can directly sense the change in climate factors and determine the onset of spring phenology, indicated by the arrows from each climate factor directly point to the SOS. Also, they can indirectly influence spring phenology by altering the photosynthetic carbon assimilation, indicated by the arrows from each climate factor firstly directly point to GPP or GPP_max_ then to the SOS. The piecewise SEM was fit using the “piecewiseSEM” package^72^ in R^68^.

Further, we quantified and compared effects of these climate variables and GPP_max_ on spring phenology using random-forest algorithm, an ensemble statistical learning method^73^ that has been frequently applied in ecological modeling and prediction^74,75^. By this, we select the most important variables which may affect forest phenology, including GPP_max_, temperature, precipitation, radiation, CO_2,_ and soil water content in FLUXNET between 1992 and 2014. We conducted random forests analysis using “randomForest” package^76^ in R^68^, where the number of trees (ntree) and variables randomly sampled as candidates (mtry) at each split were set to 1000 and 4 respectively to guarantee the reliability of the result^77^. These same analyses were repeated using average GPP in place of GPP_max_.

All data analyses were conducted using R version 4.0.3^68^.

### Reporting summary

Further information on research design is available in the Nature Research Reporting Summary linked to this article.

## Supplementary information

Supplementary information

Reporting Summary

## Data Availability

The PEP725 phenological data was accessed from www.pep725.eu, PhenoCam phenological data was obtained from https://phenocam.sr.unh.edu/, GIMMS NDVI3g dataset was downloaded from http://ecocast.arc.nasa.gov, FLUXNET dataset was downloaded from https://fluxnet.org/data/. Climate data were downloaded from E-OBS (http://ensembles-eu.metoffice.com), World Meteorological Organization (http://climexp.knmi.nl/select.cgi?id=someone@somewhere&field=clm_wfdei_soil01) and Climate Research Unit (https://crudata.uea.ac.uk/cru/data/hrg/cru_ts_4.04/; http://www.geodata.cn).

## Code availability

The primary codes used in this study are available at https://figshare.com/articles/software/code_information/20037647.
