# Author Correction: Warming-induced increase in carbon uptake is linked to earlier spring phenology in temperate and boreal forests

**DOI:** 10.1038/s41467-022-31972-3

**Published:** 2022-07-18

**Authors:** Hongshuang Gu, Yuxin Qiao, Zhenxiang Xi, Sergio Rossi, Nicholas G. Smith, Jianquan Liu, Lei Chen

**Affiliations:** 1 Key Laboratory of Bio-Resource and Eco-Environment of Ministry of Education, College of Life Sciences, Sichuan University, Chengdu, China; 2 Département des Sciences Fondamentales, Université du Québec à Chicoutimi, Chicoutimi, QC Canada; 3 Department of Biological Sciences, Texas Tech University, Lubbock, TX USA

**Keywords:** Phenology, Forest ecology

Correction to: *Nature Communications* 10.1038/s41467-022-31496-w, published online 27 June 2022.

The original version of this Article contained errors in Fig. 1 and in the legend of Fig. 3. In Fig. 1, the region of Russia was inadvertently omitted from the background map layer. In the legend of Fig. 3, the description of the panels incorrectly read “**a** The S based upon PhenoCam data in deciduous broadleaf and evergreen needleleaf forests. **b** The S based upon GIMMS NDVI3g data in boreal and temperate forests.” The correct version states ‘S_T_’ in place of ‘S’. These have been corrected in both the PDF and HTML versions of the article.

