# Supplementary material for: Warming-induced increase in carbon uptake is linked to earlier spring phenology in temperate and boreal forests
Source: Nat Commun. 2022 Jun 27;13:3698. doi: 10.1038/s41467-022-31496-w (PMC9237039; doi:10.1038/s41467-022-31496-w)
Supplement: Supplementary file 1 — Supplementary information [file 41467_2022_31496_MOESM1_ESM.pdf]

Supplementary Information for

Warming-induced increase in carbon uptake is linked to earlier spring phenology in temperate and boreal forests

This PDF file contains:
Supplementary Tables 1-6
Supplementary Figures 1-3

Supplementary Table 1. List of the nine temperate species selected from the PEP725 phenological network.

Number	Latin Name	Abbreviated Name	Common Name
1	*Aesculus hippocastanum* L.	*A. hippocastanum*	Horse Chestnut
2	*Alnus glutinosa* Medik.	*A. glutinosa*	Black Alder
3	*Betula pendula* Roth.	*B. pendula*	White Birch
4	*Ribes grossularia*	*R. grossularia*	European Gooseberry
5	*Fagus sylvatica*	*F. sylvatica*	European Beech
6	*Fraxinus excelsior* L.	*F. excelsior*	European Ash
7	*Quercus robur* Linnaeus	*Q. robur*	European Oak
8	*Tilia cordata* Mill.	*T. cordata*	Little-leaf Linden
9	*Tilia platyphyllos* Scop.	*T. platyphyllos*	Large-leaf Linden

Supplementary Table 2. Statistics on temperature sensitivities (S_T, change in days per degree Celsius) of different species or vegetation types in three phenological datasets. N indicate the sample size; CI indicate the confidence interval.

Dataset	Species/Vegetation Type	Mean S_T	N	(95%) CI
PEP725	*Aesculus hippocastanum* L.	-0.24	1956	(-0.24, -0.23)
PEP725	*Alnus glutinosa* (L.) Gaertn.	-0.24	936	(-0.24, -0.23)
PEP725	*Betula pendula* Roth	-0.23	1940	(-0.23, -0.22)
PEP725	*Fagus sylvatica* L.	-0.35	1467	(-0.35, -0.34)
PEP725	*Fraxinus excelsior* L.	-0.29	979	(-0.29, -0.28)
PEP725	*Quercus robur* L.	-0.31	1570	(-0.31, -0.30)
PEP725	*Ribes grossularia* L.	-0.20	1646	(-0.20, -0.19)
PEP725	*Tilia cordata* Mill.	-0.09	351	(-0.09, -0.08)
PEP725	*Tilia platyphyllos* Scop.	-0.09	551	(-0.09, -0.08)
PhenoCam	Deciduous Broadleaf	-0.08	57	(-0.14, -0.03)
PhenoCam	Evergreen Needleleaf	-0.17	18	(-0.27, -0.07)
GIMMS $NDVI_{3g}$	Boreal Forests/Taiga	-0.11	3746	(-0.12, -0.10)
GIMMS $NDVI_{3g}$	Temperate Broadleaf & Mixed Forests	-0.14	167	(-0.17, -0.11)
GIMMS $NDVI_{3g}$	Temperate Conifer Forests	-0.11	1508	(-0.13, -0.10)

Supplementary Table 3. Statistics of Tukey's multiple comparisons of different species or vegetation types in three phenological datasets. CI indicate the confidence interval. The two-sided test was used to calculate *P* values.

Dataset	Species/Vegetation Types		Difference	(95%) CI		*P* Value
				Lower	Upper	
PEP725	*Alnus glutinosa* Medik.	*Aesculus hippocastanum* L.	0.00	-0.02	0.03	1.00
PEP725	*Betula pendula* Roth.	*Aesculus hippocastanum* L.	0.01	0.00	0.03	0.21
PEP725	*Fagus sylvatica*	*Aesculus hippocastanum* L.	0.11	-0.13	-0.09	<0.001
PEP725	*Fraxinus excelsior* L.	*Aesculus hippocastanum* L.	-0.05	-0.07	-0.03	<0.001
PEP725	*Quercus robur* Linnaeus	*Aesculus hippocastanum* L.	-0.07	-0.09	-0.05	<0.001
PEP725	*Ribes grossularia*	*Aesculus hippocastanum* L.	0.05	0.03	0.07	<0.001
PEP725	*Tilia cordata* Mill.	*Aesculus hippocastanum* L.	0.15	0.12	0.18	<0.001
PEP725	*Tilia platyphyllos* Scop.	*Aesculus hippocastanum* L.	0.15	0.12	0.18	<0.001
PEP725	*Betula pendula* Roth.	*Alnus glutinosa* Medik.	0.01	-0.01	0.03	0.82
PEP725	*Fagus sylvatica*	*Alnus glutinosa* Medik.	-0.11	-0.14	-0.09	<0.001
PEP725	*Fraxinus excelsior* L.	*Alnus glutinosa* Medik.	-0.05	-0.08	-0.03	<0.001
PEP725	*Quercus robur* Linnaeus	*Alnus glutinosa* Medik.	-0.07	-0.10	-0.05	<0.001
PEP725	*Ribes grossularia*	*Alnus glutinosa* Medik.	0.04	0.02	0.07	<0.001
PEP725	*Tilia cordata* Mill.	*Alnus glutinosa* Medik.	0.15	0.11	0.18	<0.001
PEP725	*Tilia platyphyllos* Scop.	*Alnus glutinosa* Medik.	0.15	0.12	0.18	<0.001
PEP725	*Fagus sylvatica*	*Betula pendula* Roth.	-0.12	-0.14	-0.10	<0.001
PEP725	*Fraxinus excelsior* L.	*Betula pendula* Roth.	-0.06	-0.09	-0.04	<0.001
PEP725	*Quercus robur* Linnaeus	*Betula pendula* Roth.	-0.08	-0.10	-0.06	<0.001
PEP725	*Ribes grossularia*	*Betula pendula* Roth.	0.03	0.01	0.05	<0.001
PEP725	*Tilia cordata* Mill.	*Betula pendula* Roth.	0.14	0.10	0.17	<0.001
PEP725	*Tilia platyphyllos* Scop.	*Betula pendula* Roth.	0.13	0.11	0.16	<0.001
PEP725	*Fraxinus excelsior* L.	*Fagus sylvatica*	0.06	0.04	0.08	<0.001

PEP725	*Quercus robur* Linnaeus	*Fagus sylvatica*	0.04	0.02	0.06	<0.001
PEP725	*Ribes grossularia*	*Fagus sylvatica*	0.15	0.13	0.17	<0.001
PEP725	*Tilia cordata* Mill.	*Fagus sylvatica*	0.26	0.23	0.29	<0.001
PEP725	*Tilia platyphyllos* Scop.	*Fagus sylvatica*	0.26	0.23	0.28	<0.001
PEP725	*Quercus robur* Linnaeus	*Fraxinus excelsior* L.	-0.02	-0.04	0.00	0.17
PEP725	*Ribes grossularia*	*Fraxinus excelsior* L.	0.10	0.07	0.12	<0.001
PEP725	*Tilia cordata* Mill.	*Fraxinus excelsior* L.	0.20	0.17	0.24	<0.001
PEP725	*Tilia platyphyllos* Scop.	*Fraxinus excelsior* L.	0.20	0.17	0.23	<0.001
PEP725	*Ribes grossularia*	*Quercus robur* Linnaeus	0.12	0.10	0.13	<0.001
PEP725	*Tilia cordata* Mill.	*Quercus robur* Linnaeus	0.22	0.19	0.25	<0.001
PEP725	*Tilia platyphyllos* Scop.	*Quercus robur* Linnaeus	0.22	0.19	0.24	<0.001
PEP725	*Tilia cordata* Mill.	*Ribes grossularia*	0.11	0.07	0.14	<0.001
PEP725	*Tilia platyphyllos* Scop.	*Ribes grossularia*	0.10	0.07	0.13	<0.001
PEP725	*Tilia platyphyllos* Scop.	*Tilia cordata* Mill.	0.00	-0.04	0.03	1.00
PhenoCam	Evergreen Needleleaf Forest	Deciduous Broadleaf Forest	0.09	-0.29	0.11	0.39
GIMMS $NDVI_{3g}$	Temperate Broadleaf & Mixed Forests	Boreal Forests/Taiga	0.03	-0.08	0.01	0.23
GIMMS $NDVI_{3g}$	Temperate Conifer Forests	Boreal Forests/Taiga	0.01	-0.02	0.01	0.72
GIMMS $NDVI_{3g}$	Temperate Conifer Forests	Temperate Broadleaf & Mixed Forests	0.03	-0.02	0.08	0.39

Supplementary Table 4. Statistics of the piecewise structural equation model (SEM) using FLUXNET data. Both climate factors (temperature, radiation, soil water content, precipitation, and CO_2) and GPP_{max} were incorporated into the SEM to explore the direct or indirect effects of climate factors and GPP_{max} on start of season (SOS). The GPP_{max} was defines as the maximum daily gross primary productivity (GPP) in each year. In the direct-effect model, the climatic factors in previous growing season were assumed to have a direct influence on SOS in current year. In the indirect-effect model, the climatic factors were assumed to influence SOS in current year by altering GPP in previous growing season. We calculated the adjusted coefficients of predictors (R^2) in each model. The value of standardized direct effect represents the effect of the predictors on the responses. The two-sided test was used to calculate *P* values.

**Overall Fit**	**Response**	**Predictor**	**Standardized Direct Effect**	***P* Value**
	GPP_{max}	Temperature	0.33	<0.001
	GPP_{max}	Soil Water Content	0.35	<0.001
R^2 = 0.24	GPP_{max}	Radiation	-0.03	0.61
	GPP_{max}	Precipitation	0.10	0.10
	GPP_{max}	CO_2	-0.05	0.39
	SOS	GPP_{max}	-0.50	<0.001
	SOS	Temperature	0.36	<0.001
R^2 = 0.23	SOS	Soil Water Content	0.18	0.01
	SOS	Radiation	-0.13	0.02
	SOS	Precipitation	-0.12	0.06
	SOS	CO_2	-0.05	0.47
AIC			30.00	
BIC			83.00	

Supplementary Table 5. Statistics of the piecewise structural equation model (SEM) using FLUXNET data. Both climate factors (temperature, radiation, soil water content, precipitation, and CO_2) and average GPP were incorporated into the SEM to explore the direct or indirect effects of climate factors and GPP on start of season (SOS). The average GPP refers to the mean daily gross primary productivity (GPP) during growing season. In the direct-effect model, the climatic factors in previous growing season were assumed to have a direct influence on SOS in current year. In the indirect-effect model, the climatic factors were assumed to influence SOS in current year by altering GPP in previous growing season. We calculated the adjusted coefficients of predictors (R^2) in each model. The value of standardized direct effect represents the effect of the predictors on the responses. The two-sided test was used to calculate *P* values.

**Overall Fit**	**Response**	**Predictor**	**Standardized Direct Effect**	***P* Value**
$R^2 = 0.22$	GPP	Temperature	0.31	<0.001
	GPP	Soil Water Content	0.35	<0.001
	GPP	Radiation	-0.05	0.39
	GPP	Precipitation	0.11	0.09
	GPP	CO_2	-0.06	0.39
$R^2 = 0.29$	SOS	GPP	-0.55	<0.001
	SOS	Temperature	0.36	<0.001
	SOS	Soil Water Content	0.20	<0.001
	SOS	Radiation	-0.15	0.01
	SOS	Precipitation	-0.11	0.07
	SOS	CO_2	-0.05	0.42
AIC			30.00	
BIC			83.00	

Supplementary Table 6. List of detailed information of the datasets used in this study.

Variable	Dataset	Time Resolution	Time Series	Note
SOS	PEP725	yearly	1951-2015	http://www.pep725.eu/
EOS	PEP725	yearly	1951-2015	http://www.pep725.eu/
SOS	PhenoCam	yearly	2000-2018	https://phenocam.sr.unh.edu/
SOS	GIMMS $NDVI_{3g}$	yearly	1982-2014	http://ecocast.arc.nasa.gov
SOS	FLUXNET	yearly	1992-2014	https://fluxnet.org/data/
CO_2	FLUXNET	daily	1992-2014	https://fluxnet.org/data/
Precipitation	FLUXNET	daily	1992-2014	https://fluxnet.org/data/
Radiation	FLUXNET	daily	1992-2014	https://fluxnet.org/data/
Soil water content	FLUXNET	daily	1992-2014	https://fluxnet.org/data/
Temperature	FLUXNET	daily	1992-2014	https://fluxnet.org/data/
GPP_{max}	FLUXNET	yearly	1992-2014	https://fluxnet.org/data/
GPP	FLUXNET	daily	1992-2014	https://fluxnet.org/data/
Humidity	E-OBS	daily	1983-2014	http://www.ecad.eu/
Precipitation	E-OBS	daily	1950-2014	http://www.ecad.eu/
Soil moisture	E-OBS	daily	1983-2014	http://www.ecad.eu/
Temperature	E-OBS	daily	1950-2014	http://www.ecad.eu/
Temperature	Climate Research Unit	monthly	1981-2017	https://crudata.uea.ac.uk/cru/data/hrg/cru_ts_4.04/cruts.2004151855.v4.04/

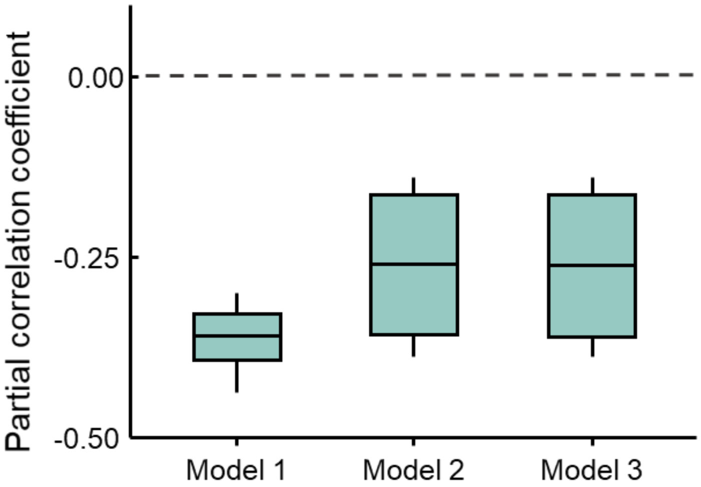

Supplementary Figure 1. Partial correlation coefficients between start of season (SOS) of current year and temperature during previous growing season (May to September) after excluding covariates based on PEP725 dataset. Leaf unfolding dates were used to represent SOS. The covariates included climate factors (radiation, precipitation, soil moisture, humidity) during growing season, autumn leaf senescence date, accumulations of chilling and forcing in winter and spring. Climate factors were excluded in Model 1, climatic factors and autumn phenology were excluded in Model 2, climatic factors, autumn leaf senescence date and chilling and forcing were excluded in Model 3. In the box plots, the box spans from the first to the third quartile, with intermediate values marked as the black line in the middle of the box. The black dash line indicates when partial correlation coefficients are equal to zero.

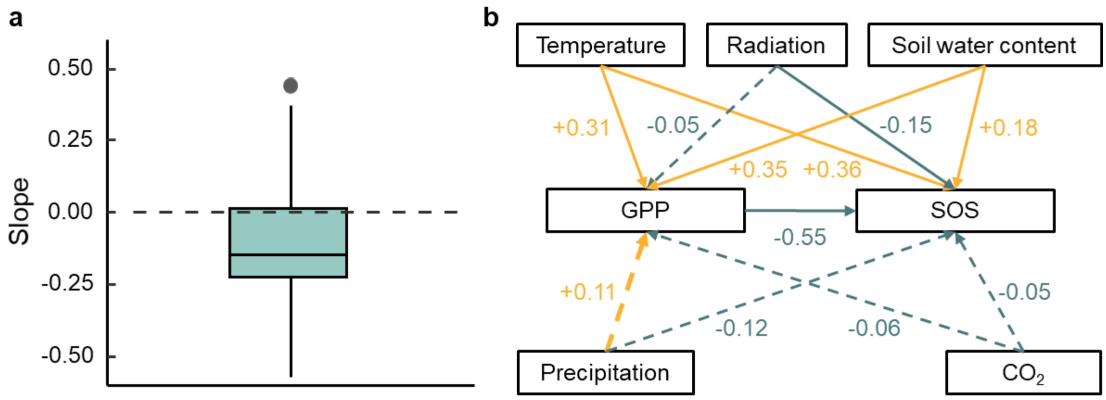

Supplementary Figure 2. **(a)** Regression coefficients (Slope) of normalized anomalies of spring phenology (SOS) in current year in response to normalized anomalies of average GPP in previous growing season (N=28). **(b)** Piecewise structural equation model (SEM) using FLUXNET data. The average GPP refers to the mean daily gross primary productivity (GPP) during growing season. In **(a)**, the black dash lines indicate when slope is equal to zero, the box spans from the first to the third quartile, with intermediate values marked as the black line in the middle of the box, and the grey points represent the outliers whose values exceed 1.5 times the length of the box. In **(b)**, both climate factors (temperature, radiation, soil water content, precipitation, and CO_2) and average GPP were incorporated into the SEM to explore the direct (arrows from each climate factor directly point to the SOS) or indirect (arrows from each climate factor firstly directly point to average GPP then to the SOS) effects of climate factors on spring phenology, with green lines indicating a negative effect and orange lines indicating a positive effect. The solid lines represent significant relationships (P<0.05) between variables, while dashed lines represent no significant relationships between variables (P>0.05). The two-sided test was used to calculate P values.

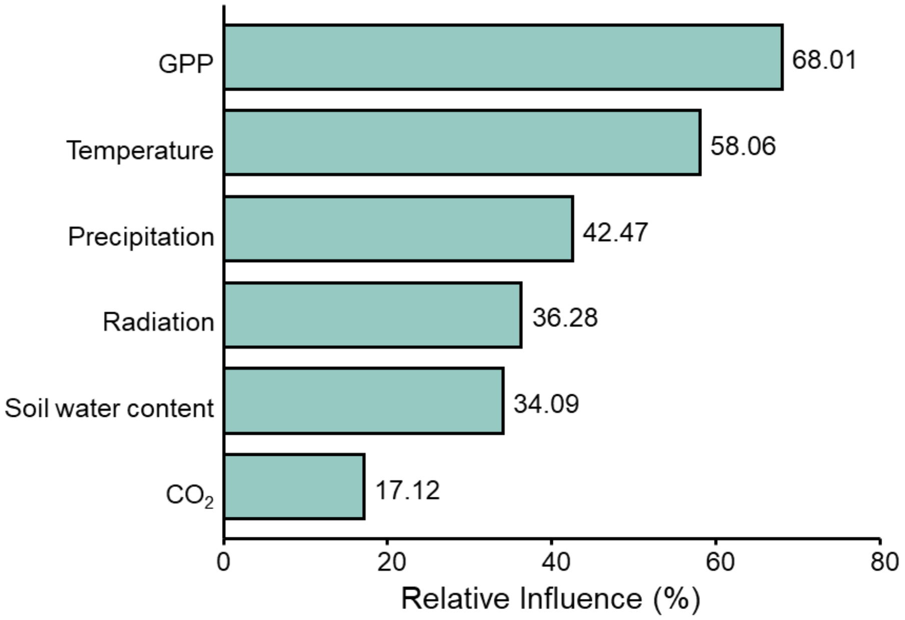

Supplementary Figure 3. Relative influence of average GPP and climatic factors during previous growing season on start of season (SOS) of current year using the data of 28 FLUXNET sites between 1992 and 2014. The average GPP refers to the mean daily gross primary productivity (GPP) during growing season. Random forest algorithm was used to quantify and compare the effects of climate variables and average GPP on SOS.
