# Supplementary material for: Warming-induced increase in carbon uptake is linked to earlier spring phenology in temperate and boreal forests
Source: Nat Commun. 2022 Jun 27;13:3698. doi: 10.1038/s41467-022-31496-w (PMC9237039; doi:10.1038/s41467-022-31496-w)
Supplement: Supplementary file 2 — Reporting Summary [file 41467_2022_31496_MOESM2_ESM.pdf]

nature portfolio

Corresponding author(s): Lei Chen

Last updated by author(s): Jun 17, 2022

Reporting Summary

Nature Portfolio wishes to improve the reproducibility of the work that we publish. This form provides structure for consistency and transparency in reporting. For further information on Nature Portfolio policies, see our Editorial Policies and the Editorial Policy Checklist.

Statistics

For all statistical analyses, confirm that the following items are present in the figure legend, table legend, main text, or Methods section.

n/a	Confirmed	
☐	☒	The exact sample size (*n*) for each experimental group/condition, given as a discrete number and unit of measurement
☐	☒	A statement on whether measurements were taken from distinct samples or whether the same sample was measured repeatedly
☐	☒	The statistical test(s) used AND whether they are one- or two-sided<br>*Only common tests should be described solely by name; describe more complex techniques in the Methods section.*
☒	☐	A description of all covariates tested
☐	☒	A description of any assumptions or corrections, such as tests of normality and adjustment for multiple comparisons
☐	☒	A full description of the statistical parameters including central tendency (e.g. means) or other basic estimates (e.g. regression coefficient) AND variation (e.g. standard deviation) or associated estimates of uncertainty (e.g. confidence intervals)
☐	☒	For null hypothesis testing, the test statistic (e.g. *F*, *t*, *r*) with confidence intervals, effect sizes, degrees of freedom and *P* value noted<br>*Give P values as exact values whenever suitable.*
☒	☐	For Bayesian analysis, information on the choice of priors and Markov chain Monte Carlo settings
☒	☐	For hierarchical and complex designs, identification of the appropriate level for tests and full reporting of outcomes
☐	☒	Estimates of effect sizes (e.g. Cohen's *d*, Pearson's *r*), indicating how they were calculated

Our web collection on statistics for biologists contains articles on many of the points above.

Software and code

Policy information about availability of computer code

Data collection	We do not use any software to collect data.
Data analysis	Linear regression was used to calculate temperature sensitivity, partial correlation analysis was used to exclude confounding effects in order to isolate the relationship between temperature and spring phenology, piecewise structural equation models (SEM) was used to analyze the relationships between climate, GPP (both average GPP and GPPmax) and SOS, random forest algorithm was used to quantify and compare effects of GPP and climate variables on spring phenology. All data analyses were conducted using R version 4.0.3.

For manuscripts utilizing custom algorithms or software that are central to the research but not yet described in published literature, software must be made available to editors and reviewers. We strongly encourage code deposition in a community repository (e.g. GitHub). See the Nature Portfolio guidelines for submitting code & software for further information.

Data

Policy information about availability of data

All manuscripts must include a data availability statement. This statement should provide the following information, where applicable:

- Accession codes, unique identifiers, or web links for publicly available datasets
- A description of any restrictions on data availability
- For clinical datasets or third party data, please ensure that the statement adheres to our policy

The PEP725 phenological data was obtained from the Pan European Phenology (PEP) network (www.pep725.eu), the PhenoCam phenological data was obtained from the PhenoCam network (https://phenocam.sr.unh.edu/) and the GIMMS NDVI3g phenological product were calculated by the third generation GIMMS NDVI3g dataset (http://ecocast.arc.nasa.gov) from Advanced Very High Resolution Radiometer (AVHRR) instruments. The FLUXNET dataset was downloaded from FLUXNET (https://fluxnet.org/data/).

Climate data were downloaded from the database E-OBS (http://ensembles-eu.metoffice.com), the World Meteorological Organization (http://climexp.knmi.nl/select.cgi?id=someone@ somewhere&field=clm_wfdei_soil01) and Climate Research Unit (https://crudata.uea.ac.uk/cru/data/hrg/cru_ts_4.04/; http://www.geodata.cn).

Field-specific reporting

Please select the one below that is the best fit for your research. If you are not sure, read the appropriate sections before making your selection.

☐ Life sciences ☐ Behavioural & social sciences ☒ Ecological, evolutionary & environmental sciences

For a reference copy of the document with all sections, see nature.com/documents/nr-reporting-summary-flat.pdf

Ecological, evolutionary & environmental sciences study design

All studies must disclose on these points even when the disclosure is negative.

Study description	This research studied the effects of climatic conditions during the growing season of previous year on spring phenology of current year. As a novel aspect, we find that the warmer temperatures in the previous growing season may influence spring phenology in current year by altering photosynthetic carbon assimilation.
Research sample	This research we focus on temperate and boreal forest, here we selected three sets of phenological data and maximumly retained the eligible records. The PEP725 phenological data with 466,988 records of nine temperate tree species at 2322 sites from 1950 to 2015 was downloaded from www.pep725.eu, PhenoCam phenological data with two vegetation types at 67 sites from 2000 to 2018 was downloaded from https://phenocam.sr.unh.edu/ and GIMMS NDVI3g phenological product with three vegetation types from 1982 to 2014 was downloaded from http://ecocast.arc.nasa.gov. The FLUXNET data was selected 28 sites with at least 10-year observations from 1992 to 2014 was downloaded from https://fluxnet.org/data/.
Sampling strategy	As for PEP725 dataset, we chose the date when the first leaf stalks were visible (BBCH11 in PEP725) to represent SOS and date when 50% leaves had their autumnal color (BBCH94 in PEP725) to represent the end of growing season (EOS). Data exceeding 2.5 times of median absolute deviation (MAD) were considered outliers and removed.<br>As for PhenoCam datasets, the 50% and 90% of the Green Chromatic Coordinate (GCC) were calculated daily to extract the date of greenness rising and falling. We selected 50% threshold of GCC_90 (GCC reaches 90th quantiles of its seasonal amplitude) as SOS, and removed outliers according to the above-mentioned procedure, and we selected sites with at least 8-year observations between 2000 and 2018.<br>As for GIMMS NDVI3g phenological product, We only kept areas outside tropics (latitudes >30 °N) that have a clear seasonal phenology53 and excluded bare lands with annual average NDVI <0.1 to reduce bias. We excluded tropical and subtropical forests, and non-forest vegetation types based on a map of terrestrial ecoregions57 and focused on northern temperate and boreal forests.<br>As for FLUXNET dataset, we focused on temperate and boreal forests in the Northern Hemisphere, we selected a total of 28 forest sites with at least 10-years of observations and > 300 daily records per year between 1992 and 2014 in the Northern Hemisphere.Singular Spectrum Analysis (SSA) filter method was first used to smooth the time series of daily GPP to minimize the noise.
Data collection	H.G. collected the PEP725 and PhenoCam data from www.pep725.eu and https://phenocam.sr.unh.edu/ separately, and clean the data according to research purpose using R version 4.0.3. Y.Q. collected GIMMS NDVI3g and FLUXNET data from http://ecocast.arc.nasa.gov and https://fluxnet.org/data/ respectively and clean the data using R version 4.0.3. H.G. downloaded the climate data from http://ensembles-eu.metoffice.com, http://climexp.knmi.nl/select.cgi?id=someone@ somewhere&field=clm_wfdei_soil01 and https://crudata.uea.ac.uk/cru/data/hrg/cru_ts_4.04/; http://www.geodata.cn and matched with phenological datasets in R version 4.0.3.
Timing and spatial scale	For PEP725 phenological data: 1950-2015; -10.22°W-26.45°E; 41.25°N-64.52°N.<br>For phenoCam phenological data: 2000-2018; -124.90°W- -68.26°W; 32.46°N-53.99°N.<br>For GIMMS NDVI3g phenological product:1982-2014; -161.62°W-167.71°E; 33.63°N-70.79°N.<br>For FLUXNET dataset:1992-2014; -121.61°W-32.92°E; 31.91°N-67.36°N.
Data exclusions	Median absolute deviation (MAD) was used to exclude outliers from the PEP725 dataset, the 50% threshold of GCC_90 (GCC reaches 90th quantiles of its seasonal amplitude) was used to select the data from phenoCam network, the Savizky-Golay filter was used to exclude outliers from the GIMMS NDVI3g dataset, the processing pipeline was used to reduce uncertainty by improving the data quality control of FLUXNET dataset. In addition, only temperate and boreal forests included in our datasets.
Reproducibility	Both the phenology and climate data are open access. The findings of our study can be reproduced using the statistical methods shown in the manuscript.
Randomization	Because the analyzed data in our study were obtained from open-access database instead of designed experiments, randomization is not applicable to our study.
Blinding	Because the analyzed data in our study were obtained from open-access database instead of designed experiments, blinding is not applicable to our study.

Did the study involve field work? ☐ Yes ☒ No

Reporting for specific materials, systems and methods

We require information from authors about some types of materials, experimental systems and methods used in many studies. Here, indicate whether each material, system or method listed is relevant to your study. If you are not sure if a list item applies to your research, read the appropriate section before selecting a response.

Materials & experimental systems

n/a	Involved in the study
☒	☐ Antibodies
☒	☐ Eukaryotic cell lines
☒	☐ Palaeontology and archaeology
☒	☐ Animals and other organisms
☒	☐ Human research participants
☒	☐ Clinical data
☒	☐ Dual use research of concern

Methods

n/a	Involved in the study
☒	☐ ChIP-seq
☒	☐ Flow cytometry
☒	☐ MRI-based neuroimaging
